# Construction of a TF–miRNA–gene feed-forward loop network predicts biomarkers and potential drugs for myasthenia gravis

**DOI:** 10.1038/s41598-021-81962-6

**Published:** 2021-01-28

**Authors:** Chunrui Bo, Huixue Zhang, Yuze Cao, Xiaoyu Lu, Cong Zhang, Shuang Li, Xiaotong Kong, Xiaoming Zhang, Ming Bai, Kuo Tian, Aigul Saitgareeva, Gaysina Lyaysan, Jianjian Wang, Shangwei Ning, Lihua Wang

**Affiliations:** 1 Department of Neurology, The Second Affiliated Hospital, Harbin Medical University, Harbin, 150081 Heilongjiang People’s Republic of China; 2 Department of Neurology, Peking Union Medical College Hospital, Chinese Academy of Medical Sciences, Beijing, 100730 People’s Republic of China; 3 Department of Head and Neck Surgery, The Third Affiliated Hospital, Harbin Medical University, Harbin, 150000 Heilongjiang People’s Republic of China; 4 Department of Neurology, Bashkir State Medical University, Bashkir, Russia; 5 College of Bioinformatics Science and Technology, Harbin Medical University, Harbin, 150081 Heilongjiang People’s Republic of China

**Keywords:** Data mining, Neurological disorders

## Abstract

Myasthenia gravis (MG) is an autoimmune disease and the most common type of neuromuscular disease. Genes and miRNAs associated with MG have been widely studied; however, the molecular mechanisms of transcription factors (TFs) and the relationship among them remain unclear. A TF–miRNA–gene network (TMGN) of MG was constructed by extracting six regulatory pairs (TF–miRNA, miRNA–gene, TF–gene, miRNA–TF, gene–gene and miRNA–miRNA). Then, 3/4/5-node regulatory motifs were detected in the TMGN. Then, the motifs with the highest Z-score, occurring as 3/4/5-node composite feed-forward loops (FFLs), were selected as statistically significant motifs. By merging these motifs together, we constructed a 3/4/5-node composite FFL motif-specific subnetwork (CFMSN). Then, pathway and GO enrichment analyses were performed to further elucidate the mechanism of MG. In addition, the genes, TFs and miRNAs in the CFMSN were also utilized to identify potential drugs. Five related genes, 3 TFs and 13 miRNAs, were extracted from the CFMSN. As the most important TF in the CFMSN, MYC was inferred to play a critical role in MG. Pathway enrichment analysis showed that the genes and miRNAs in the CFMSN were mainly enriched in pathways related to cancer and infections. Furthermore, 21 drugs were identified through the CFMSN, of which estradiol, estramustine, raloxifene and tamoxifen have the potential to be novel drugs to treat MG. The present study provides MG-related TFs by constructing the CFMSN for further experimental studies and provides a novel perspective for new biomarkers and potential drugs for MG.

## Introduction

Myasthenia gravis (MG) is an autoimmune disease in which antibodies destroy acetylcholine receptors on the postsynaptic membrane of the neuromuscular junction^1^. MG and its various subtypes are the major causes that affect the neuromuscular junction^1^. With the development of high-throughput data, research on MG from protein-encoding genes to noncoding RNAs, especially miRNAs, has been growing exponentially. However, the ultimate molecular mechanisms underlying the pathogenesis of MG remain to be further explored.

Among many genetic factors, miRNAs and transcription factors (TFs) are two types of key gene regulators, and they both participate in many important cellular processes that share a common regulatory logic in the coregulation of target genes. MiRNAs mainly regulate gene expression at the posttranscriptional level, while TFs modulate gene transcription at the transcriptional level^2^. Substantial evidence has demonstrated that miRNAs participate in explaining the mechanism of MG. For example, the abnormal expression of miR-15a facilitates proinflammatory cytokine production by regulating the expression of C-X-C motif chemokine 10 (CXCL10), thereby contributing to the immune response in MG^3^. miR-139-5p and miR-452-5p were approved to negatively regulate regulator of G protein signalling 13 (RGS13) expression by assessing the miRNA and mRNA profiles of the MG thymus^4^. Moreover, it has been discovered that dysregulation of TFs, which leads to significant alterations in gene expression, is a novel phenomenon in MG. For instance, Yong et al*.* detected that altered expression of the transcription factors IRF4 and IRF8 is crucial for the counterbalancing mechanisms controlling the differentiation of plasma cells in patients with MG^5^. Li et al*.* verified that the overexpression of FRA1, also known as FOSL1, a FOS member of the activator protein 1 (AP-1) transcription factor, disrupted inflammatory cytokine secretion by medulla thymic epithelial cells (mTECs) in the MG thymus^6^. Nevertheless, as gene regulators, how miRNAs and TFs cooperate to regulate gene expression to cause the pathogenesis of MG has not yet been investigated.

Recently, comprehensive analyses have been proposed to elucidate these gene regulators (miRNAs and TFs) as motifs, such as the feed-forward loop (FFL), which consists of two regulators, one of which regulates the other, and both collectively regulate a target gene^7^. It has been reported that FFLs can form recurrent network motifs to enhance the robustness of gene regulation in mammalian genomes^8^. The primary type was a three-node FFL consisting of a miRNA, a TF, and a common gene target^7,9^. A three-node FFL could be extended to generate a four-node FFL, and a coexpressed gene was added on the basis of a three-node FFL as a common target of both miRNA and TF^9^. In the same way, a five-node FFL was created by introducing an additional miRNA–miRNA interaction to the existing four-node FFL^10^. According to the regulatory module between miRNA and TF, all FFLs are classified into the following three main types: miRNA FFL, TF FFL, and composite FFL^9^.

A multitude of studies have been published to elucidate the underlying molecular mechanism by analysing FFL in many human diseases. For example, Engel et al*.* revealed neuronal activity-dependent P2X7R expression, which is induced by the transcription factor Sp1 and repressed in a calcium-dependent manner by microRNA-22^11^. Shi et al*.* proposed a novel method for identifying dysregulated miRNA–TF FFLs and excavating potential biomarkers for hypertrophic cardiomyopathy (HCM)^12^. A five-node FFL network was constructed to delineate miRNA, TF, and gene interactions in ischaemic stroke, and NFKB and STAT were identified as the chief regulators to explain the underlying mechanism of ischaemic stroke^10^. However, TF–miRNA–gene FFLs of MG have not been explored. Therefore, FFLs can be used to decipher the mechanisms of MG to provide new clues for understanding the pathogenesis and improving the treatment of MG.

In this study, we designed a network-based analysis pipeline to decipher the potential mechanism and underlying drugs for MG, which is summarized in Supplementary Fig. S1 online. First, a TF–miRNA–gene network of MG was constructed by extracting six regulatory pairs (TF–miRNA, miRNA–gene, TF–gene, miRNA–TF, gene–gene, and miRNA–miRNA) from several public databases. Then, 3-node, 4-node and 5-node regulatory motif types were detected in the network. Following the criteria we set to define 3-node, 4-node and 5-node motifs, the motif with the highest Z-score (3-node composite FFL, 4-node composite FFL and 5-node composite FFL) was selected as the statistically significant motif. By merging these motifs together, we constructed a 3/4/5-node composite FFL motif-specific subnetwork (CFMSN) and extracted related genes, TFs and miRNAs for further enrichment analysis. We found that the genes, TFs and miRNAs in the CFMSN were mainly enriched in cancerous and infectious pathways. In addition, the genes, TFs and miRNAs in the CFMSN were also applied to identify potential drugs to greatly improve the treatment of MG. Therefore, this study provides a novel perspective on the pathogenesis and treatment of MG.

## Results

### Construction of a global view of the TF–miRNA–gene network for MG

By analysing the six regulatory relationships (miRNA–TF, miRNA–gene, TF–miRNA, TF–gene, gene–gene and miRNA–miRNA), we discovered several peculiarities. Among 276 miRNA–gene pairs, 82 MG risk genes were found to be validated targets for 73 MG risk miRNAs. Among 73 miRNAs, hsa-miR-125b-5p targeted the most genes, while VEGFA was the top target gene of miRNAs. Other top miRNAs were hsa-miR-145-5p and hsa-miR-29b-3p, while the top target genes were BCL2, IGF1R and MYC. Of 39 miRNA–TF pairs, hsa-miR-29b-3p was found to target the greatest number of TFs, and MYC was targeted by the largest number of miRNAs. In addition, among 69 regulatory pairs of TF–miRNAs, the transcription factor MYC was found to regulate the highest number of miRNAs, while hsa-miR-17-5p was the top regulated miRNA in the list. In addition, the transcription factor MYC was identified to target the highest number of MG risk genes, and MYC and VEGFA were the most targeted genes among 51 TF–gene pairs.

**Figure 1:**
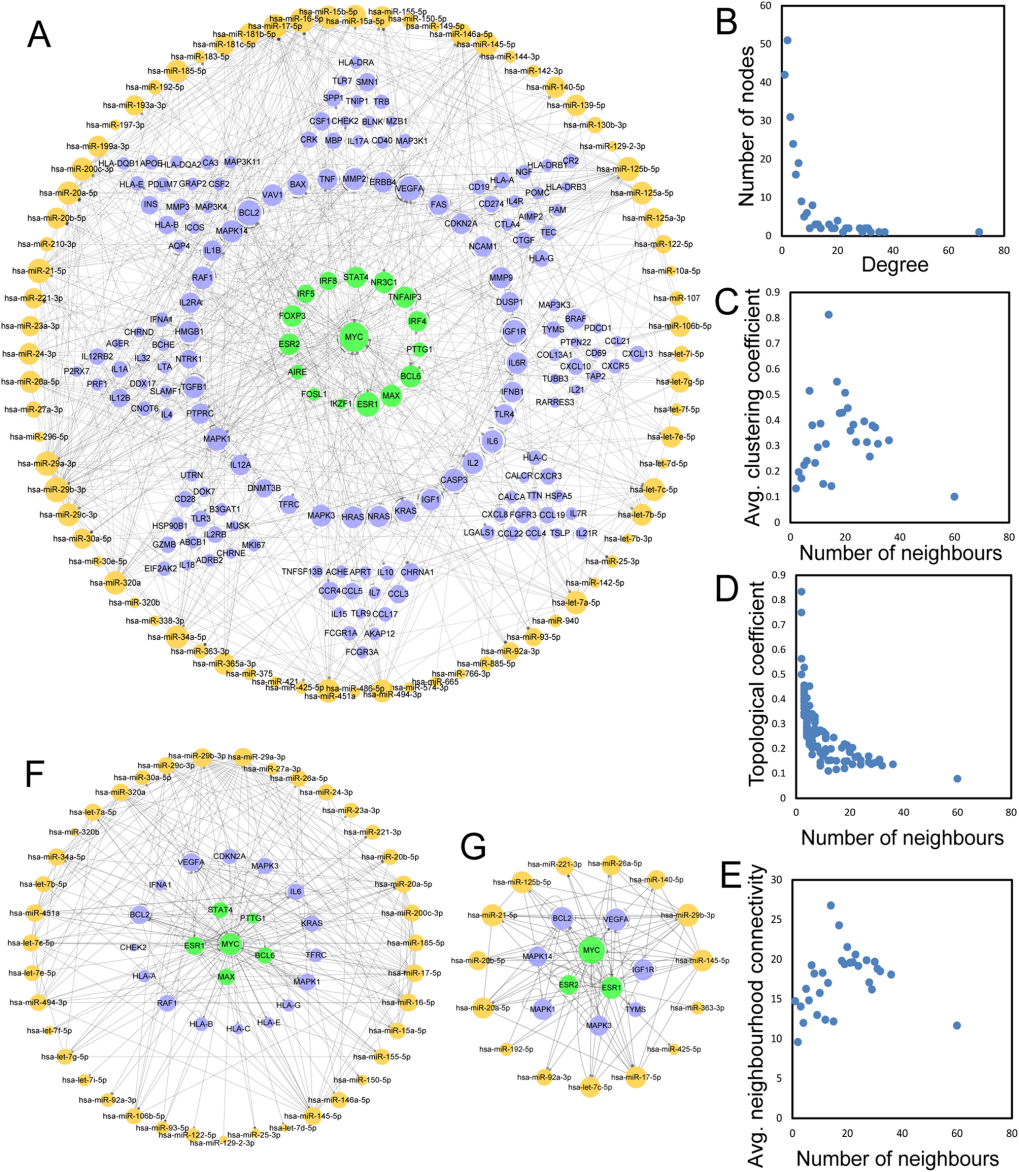
The basic characteristics of TMGN with all six types of regulatory pairs (miRNA–gene, miRNA–TF, TF–miRNA, TF–gene, gene–gene and miRNA–miRNA). (**A**) A global of the network of TMGN. TFs, miRNAs and genes are colored green, orange and blue, respectively. (**B**–**E**) The basic features of the network include degrees, clustering coefficients, topological coefficients and neighborhood connectivity of the network. (**F**) A sub-network with MYC as the central node. (**G**) A sub-network with ESR1 as the central node. TMGN: TF–miRNA–gene network; TF: transcription factor.

We constructed a global view of the TF–miRNA–gene network (TMGN) for MG by merging the six regulatory relationships (miRNA–TF, miRNA–gene, TF–miRNA, TF–gene, gene–gene and miRNA–miRNA) identified above, and the network is visualized in Fig. 1A. The network included 248 nodes (16 TFs, 76 miRNAs and 156 genes) and 799 edges. Among these edges, 276 belonged to miRNA–gene pairs, 39 belonged to miRNA–TF pairs, 47 belonged to TF–gene pairs, 60 belonged to TF–miRNA pairs, 222 belonged to gene–gene pairs, and 194 belonged to miRNA–miRNA pairs.

To examine the global view of the TMGN, we first analysed the topological features of this network, including degrees, clustering coefficients, topological coefficients and neighbourhood connectivity (Fig. 1B–E). We observed that a majority of nodes had a low degree, and only a few nodes had a high degree. Like many other biological networks, the degree distribution of this TMGN displayed a power law distribution f(x) = 78.338x^(− 1.177) with an R^2^ of 0.873, indicating that TMGN followed a scale-free distribution and presented a small-world phenomenon^13^. The nodes with a larger degree are often network hubs and are considered to play important roles in maintaining the overall connectivity of the network^14^. In the TMGN, MYC and ESR1 had the top two largest numbers of transcription factors, so we generated subnetworks with MYC and ESR1 as the central node and their first neighbours (Fig. 1F,G); MYC was found to be connected to 5 TF&genes, 16 genes and 39 miRNAs, while ESR1 was connected to 2 TF&genes, 7 genes and 15 miRNAs. These results indicate that a global view of the TMGN could be a useful background for studies of MG.

### Detection of 3/4/5-node motifs in the TMGN and discovery of feed-forward loops

**Figure 2:**
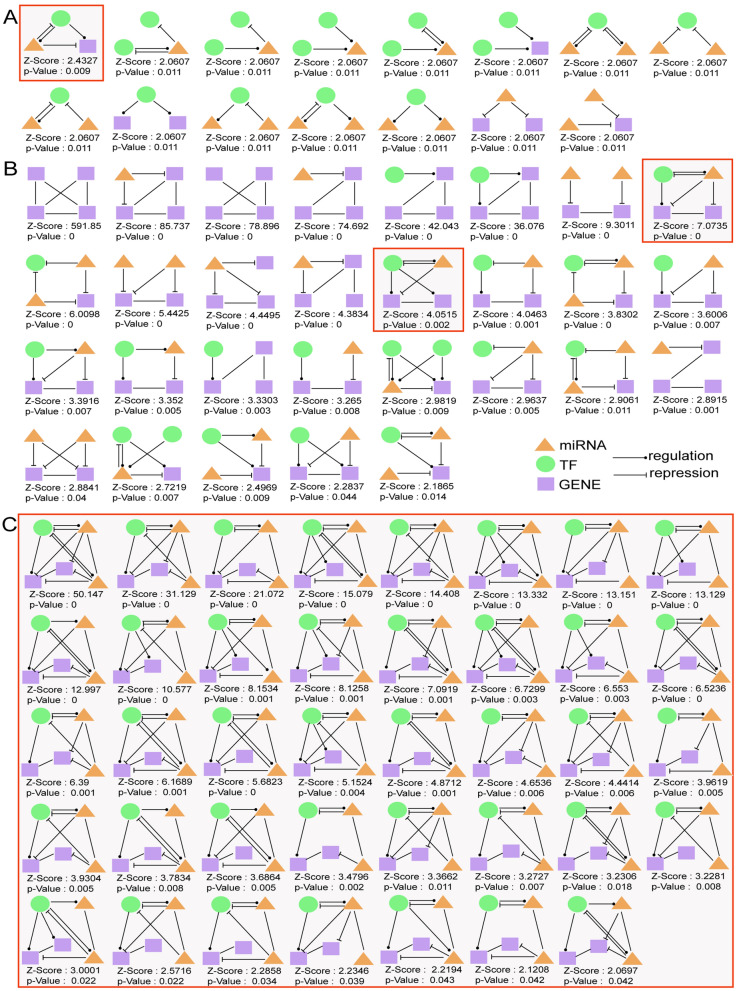
3-node, 4-node and 5 node motifs. (**A**) 15 different types of 3-node regulatory motifs. (**B**) 29 different types of 4-node regulatory motifs. (**C**) 39 screened different types of 5-node regulatory motifs. The motifs are composed of miRNAs, TFs, and target genes and their Z-scores and *p* value s are presented. Orange triangles represent miRNAs, green rounds represent TFs and purple squares represent genes. An arrow ending in a circle represents the regulatory relationship, while, an arrow ending in a “T” represents the repression relationship. Six types of relationships involved in these motifs: miRNA–gene (miRNA represses gene expression); miRNA–TF (miRNA represses TF gene expression); TF–miRNA (TF regulates miRNA expression); TF–gene (TF regulates gene expression); gene–gene (gene and gene interaction); and miRNA–miRNA (miRNA and miRNA interaction). Motifs surrounded by red squares (3-node, 4-node and 5-node composite feed-forward loop) as significant motifs were merged to form 3-node, 4-node and 5-node regulatory sub-networks, respectively. TF: transcription factor; FFL: feed-forward loop.

By combining the miRNA–TF, miRNA–gene, TF–miRNA, TF–gene, gene–gene and miRNA–miRNA interactions, a TF–miRNA–gene network (TMGN) was constructed. Then, 3-node regulatory motifs were detected in the resulting network (TMGN) with four regulatory pairs. Here, we opted to investigate only motif types having a Z-score higher than 2 and *p* value less than 0.05. As a result, 15 different types of 3-node network motifs were identified using the FANMOD tool, and the results are visualized in Fig. 2A. Following the definition of 3-node motifs and compared with other motif types, the three-node composite FFL (miRNA–TF, miRNA–gene, TF–miRNA, and TF–gene) motif (surrounded by a red square in Fig. 2A) was selected as the statistically significant motif (Z-score: 2.4327, *p* value: 0.009).

Similarly, 4-node regulatory motifs were also detected with five regulatory pairs (TF–miRNA, miRNA–gene, TF–gene, miRNA–TF, and gene–gene) using the FANMOD tool, and cut-off values of a Z-score higher than 2 and a *p* value less than 0.05 were set. As a result, a total of 29 different types of 4-node motifs were detected (shown in Fig. 2B). Following the definition of 4-node motifs, two four-node composite feed-forward loop (FFL) (TF–miRNA, miRNA–gene, TF–gene, miRNA–TF, and gene–gene) motifs (surrounded by red squares in Fig. 2B) met the criteria, and the motif with the highest Z-score was selected as the statistically significant motif (Z-score: 7.0735, *p* value: 0).

Analogously, 5-node regulatory motifs were also detected with six regulatory pairs (TF–miRNA, miRNA–gene, TF–gene, miRNA–TF, gene–gene and miRNA–miRNA) using the FANMOD tool, and the same cut-off values of a Z-score higher than 2 and a *p* value less than 0.05 were set. As a consequence, 1775 different types of 5-node motifs were detected. Following the definition that we set for a 5-node motif, 39 different types of 5-node motifs were selected (visualized in Fig. 2C). Finally, a five-node composite feed-forward loop (FFL) (TF–miRNA, miRNA–gene, TF–gene, miRNA–TF, gene–gene and miRNA–miRNA) motif with the highest Z-score (Z-score: 50.147, *p* value: 0) was selected as the most statistically significant motif.

Motifs with the highest Z-score (3-node, 4-node and 5-node composite feed-forward loop (FFL)) were selected as significant motifs and merged to form 3-node, 4-node and 5-node regulatory subnetworks, respectively.

### Generation of a 3/4/5-node composite feed-forward loop (FFL) motif-specific subnetwork

**Figure 3:**
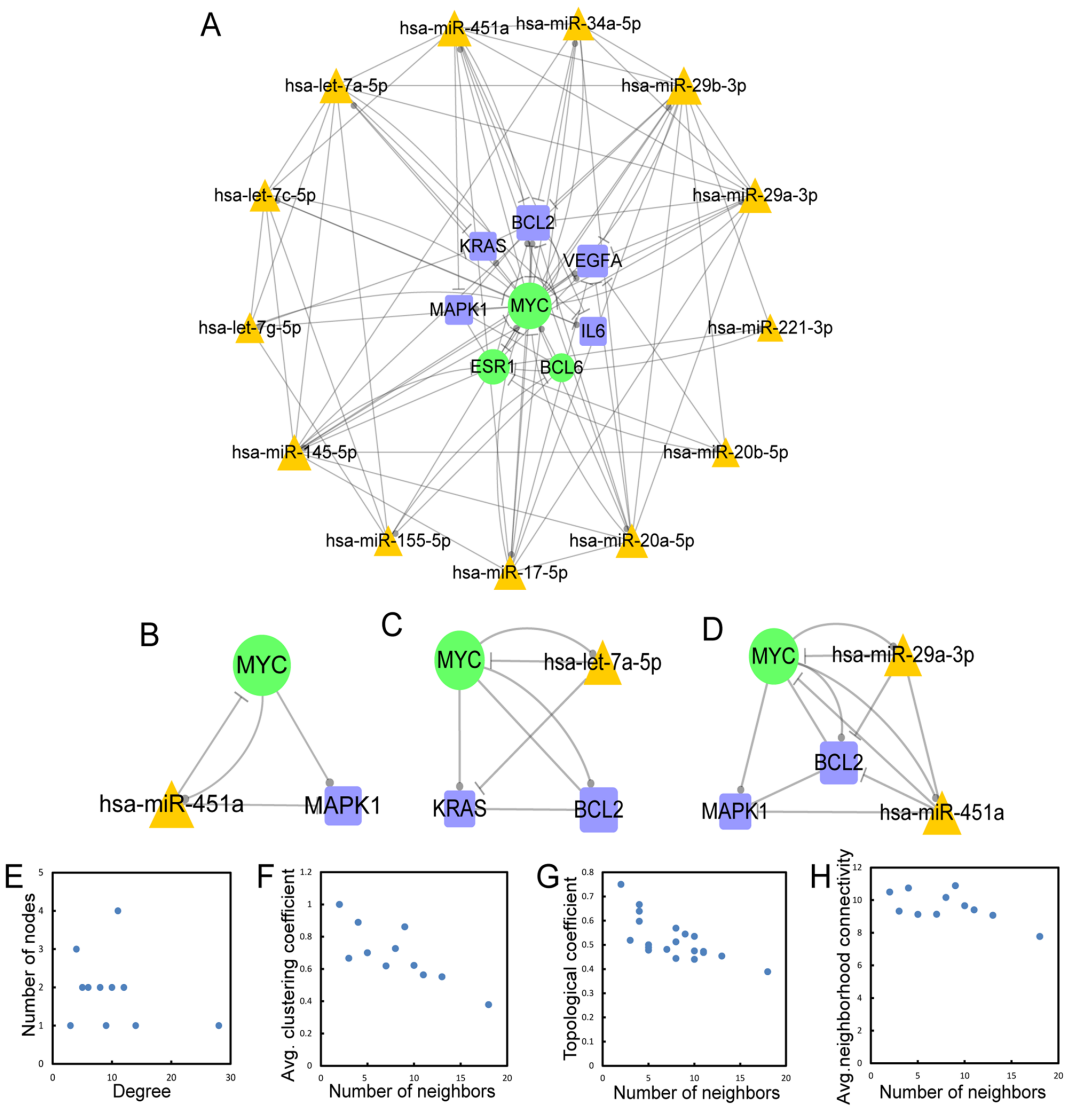
3/4/5-node composite FFL motif-specific sub-network (CFMSN). (**A**) A global view of 3/4/5-node composite FFL motif-specific sub-network (CFMSN). Orange triangle represents miRNA, green round represents TF and blue square represents gene. A line ending in a circle represents the regulatory relationship, while, a line ending in a “T” represents the repression relationship. (**B**–**D**) The examples of 3/4/5 node composite FFL. (**E**–**H**) The basic features of the network include degrees, clustering coefficients, topological coefficients and neighborhood connectivity of the network. TF: transcription factor; FFL: feed-forward loop.

The regulatory subnetwork, as well as the 3/4/5-node composite FFL motif-specific subnetwork (CFMSN), was visualized by Cytoscape 3.6.1., which is presented in Fig. 3A. We obtained 3/4/5-node FFLs with 13 miRNAs, 3 TFs, and 5 genes. The CFMSN comprised miR-20b-5p, miR-451a, miR-17-5p, miR-145-5p, miR-155-5p, miR-34a-5p, miR-20a-5p, miR-29b-5p, miR-221-5p, miR-29a-5p, let-7a-5p, let-7c-5p and let-7 g-5p as the principal miRNAs with three transcription factors (TFs), MYC, ESR1 and BCL6, and five genes, BCL2, VEGFA, KRAS, IL6 and MAPK1. Then, we extracted examples of the 3-node, 4-node and 5-node motifs (as well as 3/4/5-node composite FFLs) from the CFMSN separately, as shown in Fig. 3B–D. These examples are consistent with the motifs we discovered in the above results. We also analysed the topological features of the CFMSN, including degrees, clustering coefficients, topological coefficients and neighbourhood connectivity (Fig. 3E–H). MYC has the largest degree in the CFMSN, indicating that as a transcription factor, MYC plays a central role in the mechanism of MG.

### Validation of the expression of miRNAs and regulatory pairs in CFMSN

To verify the expression of the 13 miRNAs we identified through the CFMSN, differential expression analysis was performed using GEO2R with the dataset GSE103812^4^. The result of the analysis is shown in Table S1. We can conclude that all 13 miRNAs were expressed in MG thymus tissue. hsa-miR-145-5p was considered to be the differentially expressed miRNA. Since this dataset is a study of ectopic germinal centres in the thymus of MG, the expression of miRNAs differs to some extent and is for reference only. Moreover, the correlation of expressions between these relationship pairs in the CFMSN was also assessed to further evaluate the accuracy of the CFMSN. The results of the correlation analysis demonstrated that 41 out of 96 regulatory pairs (42.7%) in the CFMSN showed significant correlations, which indirectly showed that the extracted network (CFMSN) is meaningful. Detail information is shown in Table S2.

### Gene Ontology and pathway analyses of genes and miRNAs in the CFMSN

**Figure 4:**
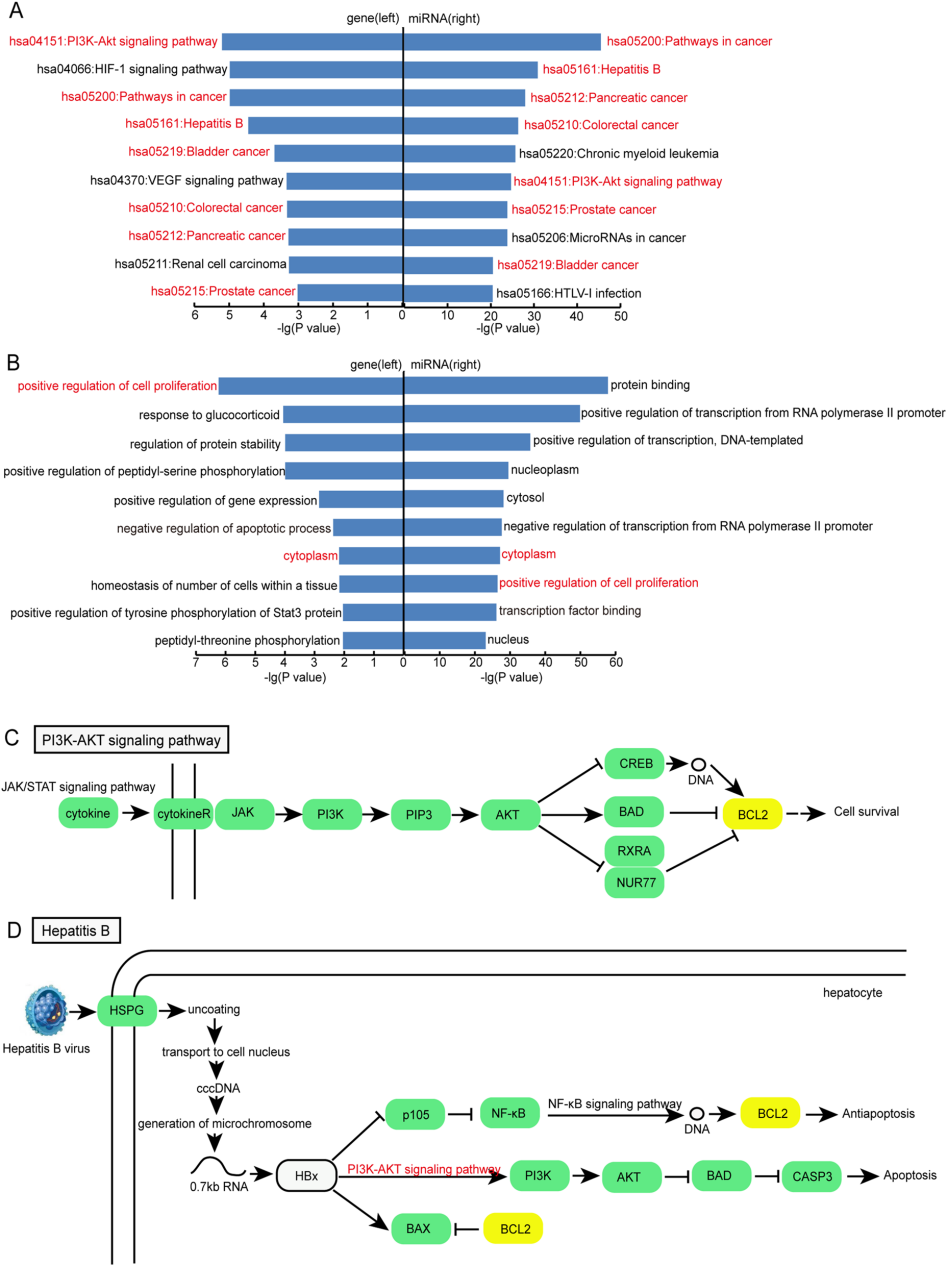
Gene Ontology and KEGG pathway analysis using genes and targets of miRNAs in CFMSN. (**A**) The top 10 pathways enriched by genes (left) and target genes of miRNAs (right). Pathways colored in red are common ones in both left and right. (**B**) The top 10 GO terms enriched by genes (left) and target genes of miRNAs (right). GO terms colored in red are common ones in both left and right. (**C**) A part of PI3K-Akt signaling pathway. (**D**) A part of Hepatitis B pathway. Genes with a yellow background is the risk genes from CFMSN. GO: Gene Ontology; KEGG: Kyoto Encyclopedia of Genes and Genomes; CFMSN: 3/4/5-node composite FFL motif-specific sub-network.

With the aid of the DAVID database, we performed Gene Ontology analysis and KEGG pathway analysis using genes and targets of miRNAs in the CFMSN. Genes that underwent enrichment analysis included MAPK1, IL6, KRAS, BCL2 and VEGFA. As a result, 41 significant KEGG pathways (*p* < 0.05) and 40 significant GO terms (*p* < 0.05) were enriched, and the top 10 pathways and GO terms are shown on the left of Fig. 4A,B. Similarly, the target genes of 13 miRNAs in the CFMSN were also enriched, and 107 KEGG pathways (*p* < 0.05) and 1127 GO terms (*p* < 0.05) were significantly enriched. The top 10 pathways and GO terms are shown on the right of Fig. 4A,B. In comparison with the left and right of Fig. 4A,B, seven pathways (PI3K-Akt signalling pathway and pathways in cancer, hepatitis B, bladder cancer, colorectal cancer, pancreatic cancer and prostate cancer) and two gene functions (positive regulation of cell proliferation and cytoplasm) were collectively enriched in both gene and miRNA analyses. Among these coenriched pathways, five were associated with cancer, and one was associated with infectious diseases, which is in accordance with our previous findings^15–17^.

Next, three significant pathways (hsa04151:PI3K-Akt signalling pathway, hsa05161: hepatitis B pathway and hsa05200:pathways in cancer) were selected for further analysis. BCL2 alone was found to be enriched in the PI3K-Akt signalling pathway (part of the pathway is shown in Fig. 4C) and hepatitis B pathway (part of the pathway is shown in Fig. 4D), taking part in cell survival and antiapoptosis. BCL2, VEGFA and IL-6 were found to be enriched in pathways in cancer. Furthermore, the PI3K-Akt signalling pathway was found to be simultaneously involved in apoptotic regulation of the hepatitis B pathway and pathways in cancer. Our results indicate that the PI3K-Akt signalling pathway may be involved in regulation of apoptosis in the pathogenesis of MG.

### Excavation of potential drugs for MG with the CFMSN

**Figure 5:**
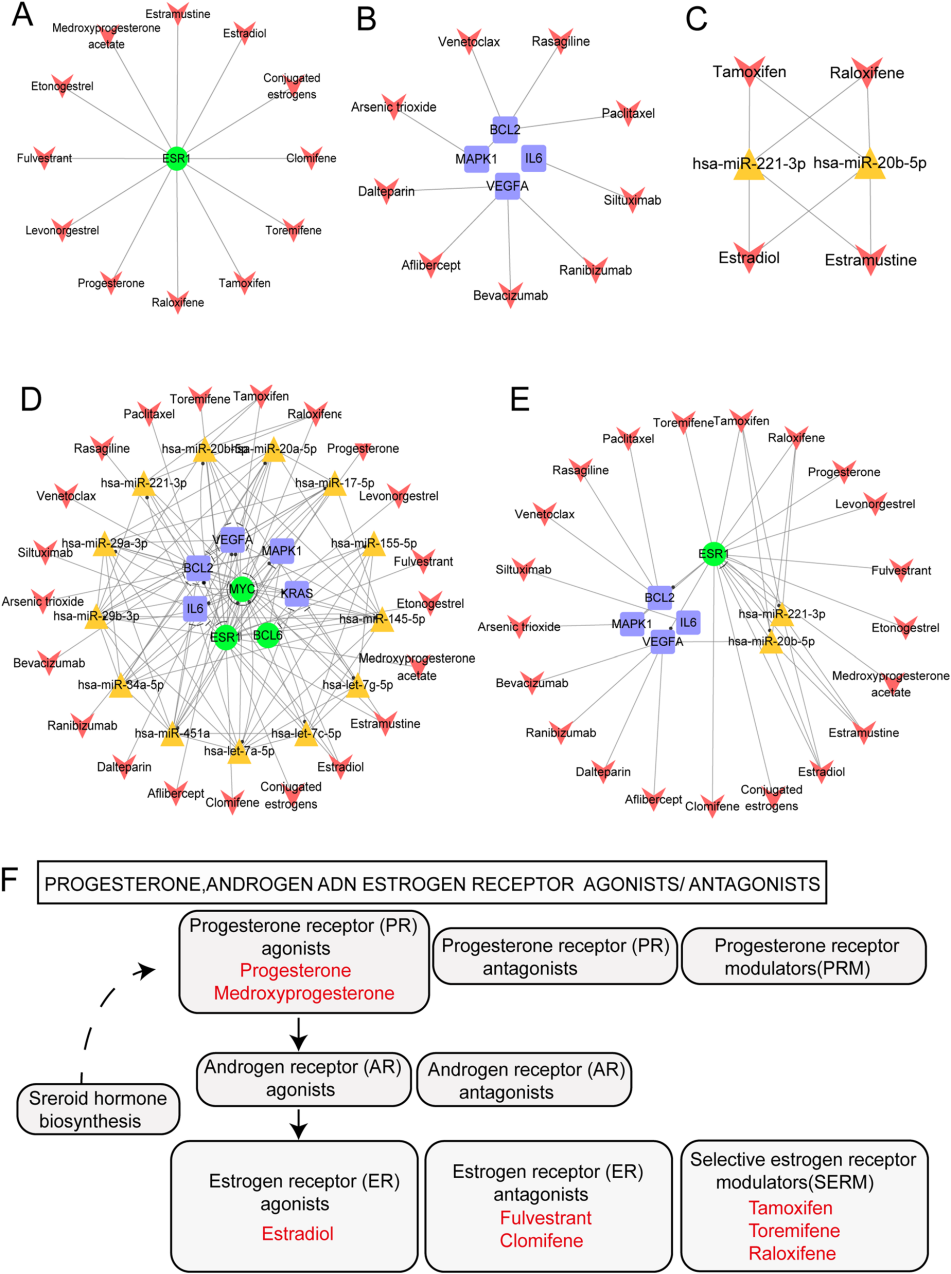
CFMSN and associated drugs. (**A**) A sub-network of TF in CFMSN and selected drugs. (**B**) A sub-network of genes in CFMSN and selected drugs. (**C**) A sub-network of miRNAs in CFMSN and selected drugs. (**D**) A global view of CFMSN and selected drugs through TFs, genes and miRNAs in CFMSN. (**E**) A sub-network of selected drugs targeted with screened TFs, genes and miRNAs. Orange triangle represents miRNA, green round represents TF, blue square represents gene and red “V” represents drug. (**F**) Part of pathway 07,226: Progesterone, androgen and estrogen receptor agonists/antagonists. Drugs colored in red are from 21 drugs we selected. CFMSN: 3/4/5-node composite FFL motif-specific sub-network; TF: transcription factor.

Based on the construction of the CFMSN, drug analysis was performed. Thus, 3 TFs, 5 genes and 13 miRNAs identified from the CFMSN were applied to discover potential drugs. The three TFs (MYC, ESR1 and BCL6) screened from the CFMSN were applied to identify potential drugs through the DrugBank database; as a result, only ESR1 was found to be targeted by 38 approved drugs. We discarded drugs that have not been approved by the FDA, are not available on the market, can lead to carcinogenesis, are illegal, have only mixed products with other drugs, are vaginally administered, or lack APRD. After screening for the above criteria, 12 drugs targeting ESR1 remained (shown in Fig. 5A).

We also identified five genes (BCL2, VEGFA, KRAS, IL6 and MAPK1) by constructing the CFMSN. By searching the DrugBank database, except KRAS, the other four out of these five genes were approved as drug targets. The drugs and their target genes are shown in Fig. 5B. These drugs are consistent with the criteria above.

The CFMSN contained miR-20b-5p, miR-451a, miR-17-5p, miR-145-5p, miR-155-5p, miR-34a-5p, miR-20a-5p, miR-29b-5p, miR-221-5p, miR-29a-5p, let-7a-5p, let-7c-5p and let-7 g-5p as the principal miRNAs. To determine how these screened miRNAs can affect the therapy and the drug targets of MG, miRNA–drug pairs were obtained by merging miRNA–gene pairs and drug-gene pairs from DrugBank. Then, we discarded the drugs with fewer than 2 target genes. A cumulative hypergeometric distribution was performed to identify significant miRNA–drug pairs. We considered results with a *p* value less than 0.05 to be statistically significant. As a result, 4 miRNAs, 13 drugs and 27 miRNA–drug pairs were identified. Only 4 drugs (estradiol, estramustine, raloxifene and tamoxifen) met the criteria we set above; thus, 2 miRNAs and 8 miRNA–drug pairs were selected (shown in Fig. 5C).

A global view of the CFMSN and selected drugs through TFs, genes and miRNAs in the CFMSN is visualized in Fig. 5D, and a subnetwork of selected drugs targeted with screened TFs, genes and miRNAs is visualized in Fig. 5E. A total of 1 TF, 2 miRNAs, 4 genes and 21 drugs were identified. Among the 21 drugs, estradiol, estramustine, raloxifene and tamoxifen had the largest degrees. Detailed information on these four drugs is summarized in Table 1.

**Table 1:** Detail information about Estradiol, Estramustine, Raloxifene and Tamoxifen.

Drug	Description	References
Estradiol	Estradiol is a naturally occurring hormone that circulates endogenously within the human body. It is the most potent form of mammalian estrogenic steroids and acts as the major female sex hormone. Estradiol plays an essential role in the regulation of the menstrual cycle, in the development of puberty and secondary female sex characteristics, as well as in ageing and several hormonally-mediated disease states	^53,54^
Estramustine	A nitrogen mustard linked to estradiol, usually as phosphate; used to treat prostatic neoplasms; also has radiation protective properties	^55,56^
Raloxifene	A second generation selective estrogen receptor modulator (SERM) used to prevent osteoporosis in postmenopausal women. It has estrogen agonist effects on bone and cholesterol metabolism but behaves as a complete estrogen antagonist on mammary gland and uterine tissue	^57–59^
Tamoxifen	One of the selective estrogen receptor modulators (SERM) with tissue-specific activities for the treatment and prevention of estrogen receptor positive breast cancer. Tamoxifen acts as an anti-estrogen (inhibiting agent) in the mammary tissue, but as an estrogen (stimulating agent) in cholesterol metabolism, bone density, and cell proliferation in the endometrium	^58,60^

To further explain the relationship between the 21 drugs we identified and the oestrogen or progesterone hormone, we dissected drug pathway 07226 in the KEGG database: progesterone, androgen and oestrogen receptor agonists/antagonists (part of the pathway is shown in Fig. 5F). Among the 21 drugs, 8 drugs were involved in this pathway. Progesterone and medroxyprogesterone are progesterone receptor (PR) agonists; estradiol is an oestrogen receptor (ER) agonist; fulvestrant and clomifene are oestrogen receptor (ER) antagonists; and tamoxifen, toremifene and raloxifene are selective oestrogen receptor modulators (SERMs). Such observations have suggested that sex steroid hormones such as oestrogens or progesterone could be potential drugs to treat MG.

## Discussion

In the present study, we have for the first time, identified the underlying mechanism of MG and screened potential drugs by detecting feed-forward loops based on miRNAs, TFs and genes and by constructing a composite FFL motif-specific subnetwork (CFMSN). Our study will provide a further comprehension of the potential molecular mechanisms and an enlargement of candidate medications for MG.

Various methods have been developed to detect network motifs, such as FANMOD^18^, WaRSwap^19^ and CoMoFinder^20^. However, the FANMOD tool was selected to detect network motifs in the present study. The reasons why we chose FANMOD are listed as follows: (i) FANMOD is one of the most widely used tools and has been widely applied to many kinds of biological networks^21,22^ and is easy to operate. (ii) In comparison with CoMoFinder and FANMOD, WaRSwap is only a randomization technique that must be used in conjunction with a motif discovery tool such as FANMOD, which substantially limits its application^19^. In addition, WaRSwap could predict a much larger set of motifs; however, it tends to overestimate the significance of candidate motifs more than CoMoFinder or FANMOD^20^. (iii) The averaged computational time for small-scale coregulatory networks in FANMOD had the advantage of being relatively faster than CoMoFinder^20^. Therefore, FANMOD is the most suitable method to detect motifs in the present study.

Although the present study has some similarities with our previous studies^15–17^, for example: (i) miRNA and gene sets of MG were re-updated based on the previous studies; (ii) the same method was used for functional enrichment analysis, and immune and cancerous pathways always represent the characteristics of MG; (iii) networks were built to screen out prominent molecules. However, there are still many differences and outstanding points in the current study: (i) TFs were first introduced to form FFLs with miRNAs and genes for MG; (ii) 21 drugs screened in the current study went through a strict criteria, and compared to the 13 drugs in the previous study^16^, only one drug (Bevacizumab) overlapped. The other drugs were first discovered to have a potential impact on MG.

The CFMSN we constructed provided an overview of the explicit mechanism of MG. Among the three TFs (MYC, ESR1 and BCL6) we identified in the CFMSN, MYC has the largest degree, which may contribute to the origin of MG. MYC was found to regulate 4 out of 5 genes (BCL2, IL6, KRAS and MAPK1) and 9 out of 13 miRNAs in the CFMSN, indicating that as a TF, MYC may be located at a core position for regulating genes and miRNAs of MG. As a TF, the MYC protein has a genome-wide distribution by regulating a number of target genes. C-myc mRNA levels were significantly reduced in MG thymus, suggesting that c-myc mediated signalling was abnormal in MG thymus^23^. In addition, abnormal expression of TFs (such as CTCF and TAF1) and MYC regulated the expression of lncRNA in MG^24^. MYC was also reported to participate in various diseases by regulating downstream target genes. For instance, insulin-like growth factor 2 mRNA-binding protein-1 (IMP-1), interrelated with c-Myc, plays an upstream role of KRAS to promote survival in the pathogenesis of colon cancer^25^; MYC activation induced by IL-6 leads to downregulation of CD33 expression in CD33 + myeloma cells^26^. However, as a TF, how MYC participates in the regulation of downstream target genes of MG has not been fully elucidated. Our results will provide more insights for future biological experiments and discover more downstream genes involved in MYC regulation associated with MG. Furthermore, in the CFMSN, 13 miRNAs were identified to play significant roles through FFLs in the pathogenesis of MG. Among the 13 miRNAs, hsa-miR-29b-3p, hsa-miR-145-5p and hsa-miR-451a had the top three degrees in the CFMSN, indicating that these three miRNAs may play important roles in further studies of the pathogenesis of MG. In addition, differential expression analysis of these 13 miRNAs was performed, and hsa-miR-145-5p was considered to be the only differentially expressed miRNA among them. Wang J et al. discovered that downregulated miR-145 promotes the pathogenetic Th17 cell response in experimental autoimmune MG (EAMG) rats through biological experiments^27^. Although hsa-miR-451a and hsa-miR-29b-3p were not differentially expressed and no biological studies have yet confirmed their relationship with MG, their expression can still be detected in MG samples. In addition, they have been discovered to have a close connection with immune-related diseases; for instance, Cheng J et al. found increased expression of miR-451a in the thymus, and deficiency in miR-451a also decreased the numbers of CD4 + CD69 + and CD4 + /CD8 + T cells^28^, which provides a theoretical basis for subsequent experimental confirmation of MG.

Pathway enrichment analysis using genes and targets of miRNAs in the CFMSN provided an intuitive overview of the role of FFL in elucidating the mechanism of MG. According to the results of the enrichment analysis, we further implicated the potential significant role of the PI3K-Akt signalling pathway in MG. The PI3K-Akt signalling pathway regulates functions as diverse as cell metabolism, survival, polarity, and vesicle trafficking^29^. As a downstream signalling pathway, immune regulators usually modulate the cells or molecules of the immune system by activating the PI3K-Akt signalling pathway. For example, 17β-oestradiol (E2) can increase the expression of PD-L1, which has been speculated to play a major role in suppressing the adaptive immune system^30^ during particular events such as autoimmune diseases^31,32^ and other disease states such as hepatitis^33^, via activation of the PI3K-Akt signalling pathway^34^. By dissecting the PI3K-Akt signalling pathway in depth, we discovered that this pathway did include several MG risk genes, especially BCL2. Therefore, we speculated that the PI3K-Akt signalling pathway was one of the most important pathways in MG and will indicate a new orientation for detecting how the PI3K-AKT signalling pathway takes part in the immune mechanism of MG.

**Figure 6:**
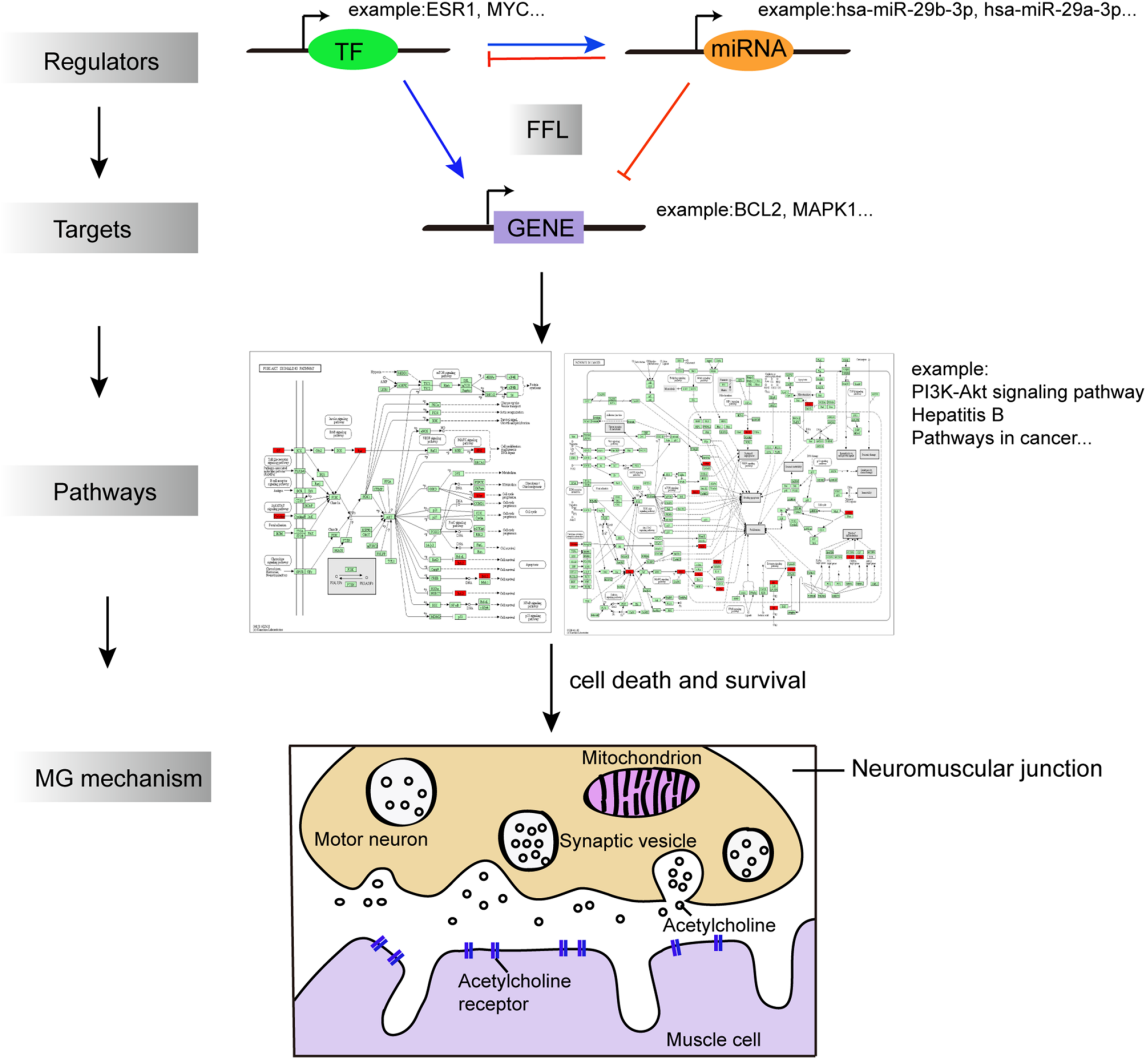
A model illustrating the misregulated processes interferred by FFLs composed of TFs, miRNAs and genes. TFs and miRNAs cooperatively mediate the pathway dysregulation which may be implicated in cell survival and apoptosis regulation mechanisms in MG. TF: transcription factor; FFL: feed-forward loop; MG: myasthenia gravis.

Here, we illustrated an example of how these regulators, including TFs and miRNAs, could explain the potential pathogenesis of MG by acting on targets and pathways (shown in Fig. 6). As shown, the transcription factor ESR1 regulates the miRNA hsa-miR-29b-3p, which in turn inhibits ESR1 expression. Furthermore, the BCL2 gene can also receive the regulation of ESR1 and the inhibition of hsa-miR-29b-3p. Thus, a feed-forward loop is formed. As a gene target, BCL2 received regulation through this FFL, which can be enriched in various pathways, such as the PI3K-AKT signalling pathway and pathways in cancer and hepatitis B, which may hint an ongoing struggle between cell death and survival mechanisms that might alter the immune response in autoimmune condition of MG.

Through the CMFSN, we screened potential drugs targeting TFs, miRNAs and genes in the network. Among the drugs we selected, we focused on four drugs: estradiol, estramustine, raloxifene and tamoxifen. It is now well known that many autoimmune diseases, including MG, are more prevalent in women than in men^35^. Furthermore, fluctuations in disease severity have been reported during pregnancy in MG patients^36^. Among these four drugs, estradiol is an oestrogen receptor (ER) agonist, estramustine is a nitrogen mustard linked to estradiol, and raloxifene and tamoxifen are selective oestrogen receptor modulators (SERMs). Delpy L et al*.* provided the first evidence that oestrogens may contribute to susceptibility to experimental autoimmune myasthenia gravis (EAMG) by promoting AChR-specific Th1 cell expansion and the development of pathogenic autoreactive B cells; this study further shed some light on the role of sex hormones in immune responses and susceptibility to autoimmune diseases in women^37^. Our results indirectly confirmed the above research and will also direct the use of either oestrogen or antiestrogen therapy in MG.

In conclusion, we systematically identified significant 3/4/5-node FFLs in MG, constructed the CFMSN, and screened potential drugs to provide new insight into potential MG treatments. Our study focused on FFLs in MG for the first time and will provide important clues for the investigation of gene regulation by miRNAs and TFs in the pathogenesis of MG. New drugs filtered through FFL networks will provide a novel future for MG treatment.

## Methods

### Acquisition of MG-related genes, miRNAs, and TFs

We obtained MG risk genes and microRNA data following previously described criteria^15–17^. MG risk genes were retrieved by searching the literature in the PubMed database (https://www.ncbi.nlm.nih.gov/pubmed) published before October 1st, 2018, following the criteria we set: the gene was notably differentially expressed in more than five MG samples using dependable biological laboratory techniques and through several databases we mentioned previously^15–17^. In addition, the catalogue of MG risk genes was updated based on our previous research^17^, and the number of MG risk genes increased from 245 to 263 (Table S3), including 131 manually collected genes (Table S4) and 132 genes from databases. MicroRNAs involved in MG were also obtained through a comprehensive literature search published before October 1st, 2018 as well as through databases such as miR2Disease^38^ (http://www.mir2disease.org/), the Nervous System Disease NcRNAome Atlas database (NSDNA)^39^ (http://www.bio-bigdata.net/nsdna/), and the Human microRNA Disease Database (HMDD v3.0)^40^ (http://www.cuilab.cn/hmdd). Detailed information on the miRNAs is shown in Table S5. Transcription factors (TFs) were retrieved from the union of 3 databases, including ChIPBase v2.0^41^ (http://rna.sysu.edu.cn/chipbase/), Transcriptional Regulatory Element Database (TRED)^42^ (http://rulai.cshl.edu/TRED) and Transcriptional Regulatory Relationships Unraveled by Sentence-based Text Mining (TRRUST v2.0)^43^ (http://www.grnpedia.org/trrust/). In addition, we selected the transcription factors present in the collected MG gene list as MG risk TFs, as shown in Table S6. As a result, 263 MG risk genes, 128 risk miRNAs and 21 risk TFs were collected. In summary, detailed information on the data set reference number, the number of MG patient samples and controls assessed, and the tissue sources of the data are listed in Tables S3–S6.

### Generation of regulatory interaction pairs among miRNAs, genes, and TFs

Six regulatory relationships (miRNA–gene, miRNA–TF, TF–miRNA, TF–gene, gene–gene, and miRNA–miRNA) were formed to generate three-, four-, and five-node motifs.

A total of 276 miRNA–gene pairs were extracted from miRTarBase^44^ (http://mirtarbase.mbc.nctu.edu.tw/), a database of experimentally validated miRNA-target interactions. miRNA–gene pairs were filtered when they showed strong evidence of interaction in humans and were in compliance with the MG gene list. Thirty-nine miRNA–TF pairs were also obtained using miRTarBase^44^, and the same procedure was used to extract the regulatory relationship between miRNAs and TFs. In other words, TFs were considered genes when finding miRNA–TF interactions.

Sixty-nine regulatory pairs of TF–miRNAs were downloaded from TransmiR v2.0^45^ (http://www.cuilab.cn/transmir), which manually collects experimentally supported TF–miRNA regulatory relationships from the literature and publications. Furthermore, 51 TF–gene pairs were acquired using TRRUST v2.0^43^ (http://www.grnpedia.org/trrust/), which contains 8,444 and 6,552 TF-target regulatory relationships among 800 human TFs and 828 mouse TFs, respectively.

A total of 224 gene–gene interaction pairs were retrieved from the Human Protein Reference Database (HPRD)^46^ (http://www.hprd.org/), a database that collects interaction networks and disease associations for each protein in the human proteome.

miRNA–miRNA interactions were established based on the common gene targets between two miRNAs^47^. Here, a common gene target means that a miRNA regulates the expression of the encoding gene of another miRNA. The miRNA–miRNA pair was selected such that there were at least two common target genes between the two miRNAs^47^; thus, 194 miRNA–miRNA pairs were acquired. In addition, the data of common target genes of miRNA–miRNA pairs were analysed, and enrichment analysis and KEGG database analysis were used to confirm the relationships among the common genes (Fig. S2). All the association information of interaction pairs is summarized in Table 2.

**Table 2:** Summary of six types of regulatory relationships among MG-related miRNAs, genes, and TFs.

Relationship	Number of pairs	Number of miRNAs	Number of genes	Number of TFs
miRNA–gene^a^	276	73	82	–
miRNA–TF^b^	39	30	–	9
TF–miRNA^c^	69	41	–	8
TF–gene^d^	51	–	35	13
gene–gene	224	–	153	–
miRNA–miRNA	194	43	–	–

*MG* myasthenia gravis, *miRNA* microRNA, *TF* transcription factor.

^a^miRNA represses the gene expression; ^b^miRNA represses the TF expression; ^c^TF regulates the miRNA expression; ^d^TF regulates the gene expression.

### Detection of 3-node, 4-node and 5-node network motifs

We first incorporated six types of regulation together to construct a TF–miRNA–gene network. These extracted regulatory relationships (TF → miRNA, miRNA → gene, TF → gene, and miRNA → TF) can be combined in different types of 3-node motifs. In addition, TF → miRNA, miRNA → gene, TF → gene, miRNA → TF, and gene–gene interaction pairs can form different types of 4-node motifs. TF → miRNA, miRNA → gene, TF → gene, miRNA → TF, gene–gene, and miRNA–miRNA pairs can formulate different types of 5-node motifs. Therefore, size 3, size 4 and size 5 network motif types in the original network were detected using FANMOD software^18^. Each motif type was evaluated for its significance using random network generation. The random networks were built 1000 times to compare with the original input network. When randomizing the network in a local constant model, edges with the same relationships were exchanged three times. Z-scores were also computed for all motif types. They indicate the frequency of motifs observed in the real network minus the mean of their occurrence in the random network divided by the standard deviation, which is the formula for calculating the Z-score. The higher the Z-score is, the more significant a motif is. Finally, from multiple types of motifs in the dumping results, 3-node, 4-node, and 5-node motif types having Z-scores > 2.0 and *p* values < 0.05 were considered significant^48^.

### Definition of 3-node, 4-node and 5-node motifs

In this article, we defined a qualified motif as a composite FFL. Therefore, we defined a 3-node motif that must include one TF, one miRNA, one gene and four types of regulatory relationships (TF → miRNA, miRNA → gene, TF → gene, and miRNA → TF), as well as a 3-node composite FFL^7,9^; a 4-node motif must include one TF, one miRNA, two genes and five types of regulatory relationships (TF → miRNA, miRNA → gene, TF → gene, miRNA → TF, and gene–gene); in other words, a qualified 4-node motif is a 3-node composite FFL with an additional gene–gene interaction pair^9^; and a 5-node motif must include one TF, two miRNAs, two genes and six types of regulatory relationships (TF → miRNA, miRNA → gene, TF → gene, miRNA → TF, gene–gene and miRNA–miRNA), that is, a qualified 5-node motif is a 4-node composite FFL with an additional miRNA–miRNA pair^9,10^.

### Motif-specific subnetwork generation and network construction

The most significant motif types (with the highest Z-score and the lowest *p* value) following the criteria we defined for 3-node, 4-node and 5-node motifs were selected to generate the subnetwork. The motif-specific subnetwork was constructed by merging motifs of the same kind together. Thus, a 3/4/5-node composite FFL motif-specific subnetwork (CFMSN) was constructed. Cytoscape software (v 3.6.1) was used for the construction of all the networks.

### Topological measurements of the network

We investigated several common topological measurements to reveal the characteristics of the networks. For the whole network, we analysed the degrees, connectivity, topological coefficients, and clustering coefficients of nodes.

### Acquisition of gene and miRNA expression profile microarray data

The microarray data were acquired from the Gene Expression Omnibus (GEO) database (www.ncbi.nlm.nih.gov/geo)^49^. The miRNA dataset GSE103812^4^, based on the GPL21572 Affymetrix Multispecies miRNA-4 Array platform, included 16 samples from the MG thymus. The gene dataset GSE103974^4^, based on GPL17586 Affymetrix Human Transcriptome Array 2.0, included 13 samples from MG thymus.

### Verification of miRNA expression levels

The miRNA dataset GSE103812^4^ was used to verify the miRNA expression levels. The interactive web tool GEO2R (www.ncbi.nlm.nih.gov/geo/geo2r)^49^ was used to verify the miRNA expression levels of samples with or without thymus germinal centres. The Benjamin and Hochberg false discovery rate (FDR) method was used to correct the adjusted *p* value and correct the occurrence of false-positive results. miRNAs were defined as differentially expressed when the *p* value < 0.05 and |logFC|> 1.

### Verify the correlation between miRNA and gene expression

The gene dataset GSE103974^4^ and miRNA dataset GSE103812^4^ were used to analyse the correlation between the regulator-target pairs. Cor.test function in R language was used to calculate the Pearson correlation coefficient of TF–miRNA, miRNA–gene, TF–gene, miRNA–TF, gene–gene and miRNA–miRNA expression in the CFMSN to assess the relevance of expressions between these regulatory relationships to further evaluate the accuracy of the network. We considered the regulatory relationship to be correlated with |cor_estimate|> 0.5 and cor_p value < 0.05.

### Functional enrichment analysis of MG genes and targets of miRNAs in the CFMSN

Pathway data were obtained from the KEGG database^50^ (https://www.kegg.jp/) to dissect several specific pathways. To identify the pathways in which MG genes and targets of miRNAs in the CFMSN were enriched, a KEGG pathway enrichment analysis was conducted by applying functional annotation tools in DAVID^51^ (https://david.ncifcrf.gov/). The significance level of KEGG pathway enrichment was calculated using a *p* value cut-off of < 0.05. Gene Ontology (GO) annotation was also performed using DAVID for the MG genes and targets of miRNAs in the CFMSN. A GO term was considered significantly enriched if it displayed a *p* value  < 0.05.

### Drugs and drug targets data

The drug and target gene data were downloaded from the DrugBank database (version 5.1.2)^52^ (https://www.drugbank.ca/). The species was limited to “*Homo sapiens*”.

### Cumulative hypergeometric distribution

We identified the significant correlations between miRNAs identified by constructing the CFMSN and drugs using cumulative hypergeometric distribution. The formula is as follows:

1 $$P = F(x{|}M,K,N) = \sum\limits_{i = 0}^{x} {\frac{{\left( {\begin{array}{*{20}c} K \\ i \\ \end{array} } \right)\left( {\begin{array}{*{20}c} {M - K} \\ {N - i} \\ \end{array} } \right)}}{{\left( {\begin{array}{*{20}c} M \\ N \\ \end{array} } \right)}}}$$

M denotes the total number of human whole genomes, K denotes the number of miRNA target genes, N denotes the number of target genes for drugs, and x represents the number of overlapping genes between miRNAs and drugs. We considered the correlation to have statistical significance if the *p* value was less than 0.05.

## Supplementary Information

Supplementary Legend.

Supplementary Figure 1.

Supplementary Figure 2.

Supplementary Table 1.

Supplementary Table 2.

Supplementary Table 3.

Supplementary Table 4.

Supplementary Table 5.

Supplementary Table 6.

## Data Availability

The datasets used and/or analyzed during the current study are available in supplemental materials.
