# Supplementary material for: Construction of a TF–miRNA–gene feed-forward loop network predicts biomarkers and potential drugs for myasthenia gravis
Source: Sci Rep. 2021 Jan 28;11:2416. doi: 10.1038/s41598-021-81962-6 (PMC7843995; doi:10.1038/s41598-021-81962-6)
Supplement: Supplementary file 1 — Supplementary Legend. [file 41598_2021_81962_MOESM1_ESM.docx]

**Figure S1. A network-based analysis pipeline deciphering the potential biomarkers and underlying drugs of MG.** MG: myasthenia gravis.

**Figure S2. Data analysis of target genes of miRNA-miRNA pairs. (A) A histogram of number of common target genes of miRNA-miRNA pairs.** The horizontal axis represents the number of common target genes of miRNA-miRNA pairs, the vertical axis represents the number of miRNA-miRNA pairs. **(B) A pie chart of the proportion of the number of target genes of miRNA-miRNA pairs. (C) A pie chart of the proportion of KEGG enrichment analysis of target genes of miRNA-miRNA pairs.**
