# Supplementary material for: Construction of a TF–miRNA–gene feed-forward loop network predicts biomarkers and potential drugs for myasthenia gravis
Source: Sci Rep. 2021 Jan 28;11:2416. doi: 10.1038/s41598-021-81962-6 (PMC7843995; doi:10.1038/s41598-021-81962-6)
Supplement: Supplementary file 4 — Supplementary Table 1. [file 41598_2021_81962_MOESM4_ESM.docx]

**Table S1. Differential expression analysis of 13 miRNAs identified in CFMSN**

| **miRNA** | **P value** | **logFC** |
| --- | --- | --- |
| hsa-miR-145-5p | 0.004773 | 1.053359 |
| hsa-let-7c-5p | 0.0312 | 0.281398 |
| hsa-miR-451a | 0.141926 | 0.855835 |
| hsa-miR-29a-3p | 0.164393 | -0.37882 |
| hsa-miR-155-5p | 0.204468 | -0.86987 |
| hsa-let-7a-5p | 0.241052 | 0.24813 |
| hsa-miR-34a-5p | 0.575468 | 0.310582 |
| hsa-miR-17-5p | 0.625406 | -0.1801 |
| hsa-miR-20a-5p | 0.685739 | -0.21349 |
| hsa-miR-29b-3p | 0.701288 | -0.23834 |
| hsa-miR-221-3p | 0.725857 | 0.095073 |
| hsa-miR-20b-5p | 0.797059 | -0.18375 |
| hsa-let-7g-5p | 0.966212 | 0.025234 |
