# Supplementary material for: Construction of a TF–miRNA–gene feed-forward loop network predicts biomarkers and potential drugs for myasthenia gravis
Source: Sci Rep. 2021 Jan 28;11:2416. doi: 10.1038/s41598-021-81962-6 (PMC7843995; doi:10.1038/s41598-021-81962-6)
Supplement: Supplementary file 5 — Supplementary Table 2. [file 41598_2021_81962_MOESM5_ESM.docx]

**Table S2. Significant expression correlations of regulatory pairs in CFMSN.**

| **node.1** | **node.2** | **edge** | **a_cor_estimate** | **a_cor_p value** |
| --- | --- | --- | --- | --- |
| **TF** | **miRNA** | **Regulatory relationship** |  |  |
| ESR1 | hsa-miR-145-5p | regulation | 0.91074691 | 0.000249 |
| BCL6 | hsa-miR-155-5p | regulation | 0.922076484 | 0.000147 |
| MYC | hsa-miR-17-5p | regulation | 0.632544164 | 0.049694 |
| ESR1 | hsa-miR-221-3p | regulation | 0.692777444 | 0.026365 |
| **miRNA** | **Gene** | **Regulatory relationship** |  |  |
| hsa-miR-155-5p | BCL6 | repression | 0.922076484 | 0.000147 |
| hsa-miR-145-5p | ESR1 | repression | 0.91074691 | 0.000249 |
| hsa-miR-221-3p | ESR1 | repression | 0.692777444 | 0.026365 |
| hsa-miR-17-5p | MYC | repression | 0.632544164 | 0.049694 |
| hsa-miR-17-5p | BCL2 | repression | 0.926989264 | 0.000114 |
| hsa-miR-20a-5p | BCL2 | repression | 0.745545178 | 0.013313 |
| hsa-miR-29a-3p | BCL2 | repression | 0.669304088 | 0.034285 |
| hsa-miR-34a-5p | BCL2 | repression | -0.845852527 | 0.002042 |
| hsa-let-7a-5p | KRAS | repression | 0.961900572 | 8.80E-06 |
| hsa-let-7g-5p | KRAS | repression | 0.845891623 | 0.00204 |
| hsa-miR-145-5p | VEGFA | repression | -0.695818458 | 0.025441 |
| **TF** | **Gene** | **Regulatory relationship** |  |  |
| ESR1 | BCL2 | regulation | 0.924685818 | 0.000128 |
| MYC | IL6 | regulation | 0.651593497 | 0.041231 |
| **Gene** | **Gene** | **Regulatory relationship** |  |  |
| MAPK1 | BCL6 | NA | 0.920768773 | 0.000157 |
| BCL2 | KRAS | NA | 0.973859862 | 1.98E-06 |
| BCL2 | MAPK1 | NA | 0.977723919 | 1.05E-06 |
| ESR1 | MAPK1 | NA | 0.851875494 | 0.001754 |
| **miRNA** | **miRNA** | **Regulatory relationship** |  |  |
| hsa-let-7a-5p | hsa-let-7c-5p | NA | 0.993712814 | 6.78E-09 |
| hsa-let-7a-5p | hsa-let-7g-5p | NA | 0.921218492 | 0.000153 |
| hsa-let-7c-5p | hsa-let-7g-5p | NA | 0.87235408 | 0.000993 |
| hsa-let-7a-5p | hsa-miR-145-5p | NA | 0.97741008 | 1.11E-06 |
| hsa-let-7c-5p | hsa-miR-145-5p | NA | 0.965234994 | 6.13E-06 |
| hsa-let-7a-5p | hsa-miR-155-5p | NA | 0.910942781 | 0.000247 |
| hsa-let-7c-5p | hsa-miR-155-5p | NA | 0.909765721 | 0.00026 |
| hsa-let-7g-5p | hsa-miR-155-5p | NA | 0.825164283 | 0.003291 |
| hsa-miR-145-5p | hsa-miR-17-5p | NA | 0.940629997 | 5.06E-05 |
| hsa-miR-145-5p | hsa-miR-20a-5p | NA | 0.843194855 | 0.002179 |
| hsa-miR-17-5p | hsa-miR-20a-5p | NA | 0.922639546 | 0.000143 |
| hsa-let-7a-5p | hsa-miR-29a-3p | NA | 0.733973184 | 0.015663 |
| hsa-miR-145-5p | hsa-miR-29a-3p | NA | 0.703104836 | 0.023317 |
| hsa-miR-17-5p | hsa-miR-29a-3p | NA | 0.803827283 | 0.005075 |
| hsa-miR-20a-5p | hsa-miR-29a-3p | NA | 0.90669083 | 0.000296 |
| hsa-miR-145-5p | hsa-miR-34a-5p | NA | -0.661482333 | 0.037246 |
| hsa-miR-17-5p | hsa-miR-34a-5p | NA | -0.643066708 | 0.044888 |
| hsa-miR-29b-3p | hsa-miR-34a-5p | NA | 0.835806968 | 0.002595 |
| hsa-miR-29b-3p | hsa-miR-451a | NA | 0.651540257 | 0.041253 |
| hsa-miR-34a-5p | hsa-miR-451a | NA | 0.77399663 | 0.008601 |
