# Supplementary material for: Construction of a TF–miRNA–gene feed-forward loop network predicts biomarkers and potential drugs for myasthenia gravis
Source: Sci Rep. 2021 Jan 28;11:2416. doi: 10.1038/s41598-021-81962-6 (PMC7843995; doi:10.1038/s41598-021-81962-6)
Supplement: Supplementary file 6 — Supplementary Table 3. [file 41598_2021_81962_MOESM6_ESM.docx]

**Table S3 A total list of MG risk genes.**

| ABCB1 | CD19 | ECD | HSPA5 | ISG20 | PDCD1 |
| --- | --- | --- | --- | --- | --- |
| ACE | CD226 | EIF2AK2 | ICOS | KCNA4 | PDLIM7 |
| ACHE | CD274 | EIF3K | IFIH1 | KRAS | POLDIP2 |
| ACP1 | CD28 | ENOX1 | IFN1@ | LGALS1 | POMC |
| ADCYAP1 | CD40 | ERBB4 | IFNA1 | LGALS8 | PRDX5 |
| ADRA1D | CD53 | ERVW-4 | IFNA13 | LIF | PRF1 |
| ADRB2 | CD55 | ESR1 | IFNB1 | LRP4 | PRSS16 |
| AGER | CD69 | ESR2 | IFNL2 | LTA | PTPN22 |
| AGRN | CD86 | FAS | IFNL3 | MAP3K1 | PTPRC |
| AHSA1 | CDKN2A | FCGR1A | IGF1 | MAP3K11 | PTTG1 |
| AIMP2 | CDR3 | FCGR2A | IGF1R | MAP3K3 | RAF1 |
| AIRE | CDS1 | FCGR3A | IGL | MAP3K4 | RARRES3 |
| AKAP12 | CFB | FCGR3B | IKZF1 | MAPK1 | RBM45 |
| AP4B1 | CHEK2 | FGFR3 | IL10 | MAPK14 | RHBDF2 |
| APOE | CHRNA1 | FOSL1 | IL12A | MAPK3 | RNF19A |
| APRT | CHRNA4 | FOXP3 | IL12B | MAX | ROBO3 |
| AQP4 | CHRNB1 | GCNT2 | IL12RB2 | MBP | SCFV |
| B3GAT1 | CHRND | GIF | IL15 | MIR145 | SEC14L3 |
| BAX | CHRNE | GPNMB | IL17A | MIR155 | SHFM1 |
| BCHE | CNOT6 | GRAP2 | IL18 | MIRLET7C | SLAMF1 |
| BCL2 | CNPY2 | GSTT1 | IL1A | MKI67 | SLC3A2 |
| BCL6 | CNTFR | GZMB | IL1B | MMP2 | SMN1 |
| BHLHE23 | COL13A1 | HAVCR1 | IL2 | MMP3 | SMN2 |
| BLNK | CORIN | HLA-A | IL21 | MMP9 | SPP1 |
| BLZF1 | CR2 | HLA-B | IL21R | MUSK | SPTLC3 |
| BRAF | CRK | HLA-C | IL25 | MYAS1 | ST2 |
| C2 | CSF1 | HLA-DOA | IL27 | MYC | STAT4 |
| CA3 | CSF2 | HLA-DPB1 | IL2RA | MYOG | TAP2 |
| CACNA1S | CTGF | HLA-DQA1 | IL2RB | MZB1 | TBC1D9 |
| CALCA | CTLA4 | HLA-DQA2 | IL32 | NCAM1 | TEC |
| CALCR | CTSS | HLA-DQB1 | IL33 | NGF | TFRC |
| CASP3 | CTSV | HLA-DQB2 | IL4 | NKX2-3 | TGFB1 |
| CAV3 | CXCL10 | HLA-DRA | IL4R | NOCT | THM |
| CCL11 | CXCL13 | HLA-DRB1 | IL6 | NR3C1 | TLR3 |
| CCL17 | CXCL8 | HLA-DRB3 | IL6R | NR4A3 | TLR4 |
| CCL19 | CXCR3 | HLA-DRB4 | IL7 | NRAS | TLR7 |
| CCL21 | CXCR5 | HLA-E | IL7R | NTRK1 | TLR9 |
| CCL22 | DDX17 | HLA-G | IL9 | ORMDL3 | TNF |
| CCL3 | DDX58 | HMGB1 | INS | OSM | TNFAIP3 |
| CCL4 | DNMT3B | HNMT | IRF4 | OVCH1 | TNFAIP8L2 |
| CCL5 | DOK7 | HRAS | IRF5 | P2RX7 | TNFRSF11A |
| CCR4 | DUSP1 | HSP90B1 | IRF8 | PAM | TNFRSF4 |
| TNFSF13B | TNIP1 | TRB | TSLP | TTN | TUBB3 |
| TYMS | UTRN | VAV1 | VEGFA | VIP |  |
