# Supplementary material for: Construction of a TF–miRNA–gene feed-forward loop network predicts biomarkers and potential drugs for myasthenia gravis
Source: Sci Rep. 2021 Jan 28;11:2416. doi: 10.1038/s41598-021-81962-6 (PMC7843995; doi:10.1038/s41598-021-81962-6)
Supplement: Supplementary file 7 — Supplementary Table 4. [file 41598_2021_81962_MOESM7_ESM.docx]

**Table S4 Information about MG risk genes (manually obtained in the literature).**

| **Gene** | **ID** | **Expression** | **Method** | **Samples** | **Reference** |  |
| --- | --- | --- | --- | --- | --- | --- |
| ERBB4 | 2066 | down regulation | microarray | 13 Patients/14 NC | 21168922 |  |
| MYC | 4609 | down regulation | RT-PCR | 8 Patients/6 NC | 11282171 |  |
| MAX | 4149 | down regulation | RT-PCR | 8 Patients/6 NC | 11282171 |  |
| ACHE | 43 | down regulation | Western blot | 9 Patients/4 NC | 17272501 |  |
| CCL21 | 6366 | up regulation | ELISA | 40 Patients/12 NC | 19847900 |  |
| CCL19 | 6363 | up regulation | ELISA | 40 Patients/12 NC | 19847900 |  |
| LGALS8 | 3964 | positive association | ELISA | 149Patients/365 NC | 22683700 |  |
| MAPK1 | 5594 | up regulation | microarray | 8 Patients/9 NC | 16272363 |  |
| MAP3K1 | 4214 | up regulation | microarray | 8 Patients/9 NC | 16272363 |  |
| MAP3K3 | 4215 | up regulation | microarray | 8 Patients/9 NC | 16272363 |  |
| MAP3K4 | 4216 | up regulation | microarray | 8 Patients/9 NC | 16272363 |  |
| MAP3K11 | 4296 | up regulation | microarray | 8 Patients/9 NC | 16272363 |  |
| BRAF | 673 | up regulation | microarray | 8 Patients/9 NC | 16272363 |  |
| NRAS | 4893 | up regulation | microarray | 8 Patients/9 NC | 16272363 |  |
| HRAS | 3265 | up regulation | microarray | 8 Patients/9 NC | 16272363 |  |
| KRAS | 3845 | up regulation | microarray | 8 Patients/9 NC | 16272363 |  |
| DUSP1 | 1843 | up regulation | microarray | 8 Patients/9 NC | 16272363 |  |
| IL6 | 3569 | up regulation | microarray | 8 Patients/9 NC | 16272363 |  |
| MAPK14 | 1432 | up regulation | microarray | 8 Patients/9 NC | 16272363 |  |
| CCL5 | 6352 | up regulation | microarray | 8 Patients/9 NC | 16272363 |  |
| GZMB | 3002 | up regulation | immunoblotting | 5 Patients/5 NC | 18675462 |  |
| HLA-A | 3105 | positive association | PCR-SSP | 109Patients/383 NC | 19490212 |  |
| HLA-B | 3106 | up regulation | PCR-SSP | 49 Patients/160 NC | 14700596 |  |
| HLA-C | 3107 | up regulation | PCR-SSP | 49 Patients/160 NC | 14700596 |  |
| HLA-DQA1 | 3117 | up regulation | Illumina HumanOmniEx-press BeadChips | 1032 Patients /1998 NC | 25643325 |  |
| HLA-DQB1 | 3119 | down regulation | PCR-SSP | 84 Patients/293 NC | 21917268 |  |
| HLA-DRB1 | 3123 | up regulation | PCR-SSP | 146Patients/250 NC | 25070808 |  |
| HLA-DRB3 | 3125 | Positive association | complement-dependent microcytotoxicity assays | 66 Patients/188 NC | 15301866 |  |
| IL32 | 9235 | up regulation | ELISA | 48 Patients/35 NC | 21487807 |  |
| TNFRSF4 | 7293 | up regulation | Flow cytometry | 36 Patients/28 NC | 16367941 |  |
| CD55 | 1604 | up regulation | sequencing | 139Patients/167 NC | 19675582 |  |
| IL2 | 3558 | up regulation | ELISA | 75 Patients/48 NC | 12646760 |  |
| IL17A | 3605 | up regulation | immunoassay | 25 Patients/14 NC | 21755509 |  |
| TLR4 | 7099 | up regulation | RT-PCR | 37 Patients/4 NC | 15972959 |  |
| CXCL13 | 10563 | up regulation | ELISA | 194 Patients/233 NC | 20223524 |  |
| CXCR5 | 643 | up regulation | immunofluorescence | 32 Patients/19 NC | 24518487 |  |
| TNF | 7124 | up regulation | PCR | 47 Patients/92 NC | 18071035 |  |
| PTPN22 | 26191 | up regulation | PCR-RFLP | 416 Patients/293 NC | 25119822 |  |
| TGFB1 | 7040 | positive association | PCR | 21 Patients/31 NC | 22458981 |  |
| IL1B | 3553 | positive association | PCR-RFLP | 107 Patients/82 NC | 9521608 |  |
| ENOX1 | 55068 | up regulation | RT-PCR | 7 Patients/764 NC | 22744667 |  |
| FCGR3B | 2215 | positive association | PCR | 30 Patients/49 NC | 9521619 |  |
| TAP2 | 6891 | positive association | PCR | 79 Patients/155 NC | 9062975 |  |
| IL4 | 3565 | down regulation | in situ hybridization | 33 Patients/25 NC | 8182116 |  |
| CNTFR | 1271 | up regulation | RT-PCR | 44 Patients/10 NC | 11694333 |  |
| CXCL10 | 3627 | up regulation | qRT-PCR | 5 Patients/6 NC | 15843529 |  |
| CXCR3 | 2833 | up regulation | qRT-PCR | 5 Patients/6 NC | 15843529 |  |
| ESR1 | 2099 | up regulation | RT-PCR | 24 Patients/8 NC | 15661863 |  |
| PRSS16 | 10279 | down regulation | RT-PCR | 12 Patients/6 NC | 15592422 |  |
| TRB | 6957 | up regulation | PCR | 34 Patients/4 NC | 14592884 |  |
| MMP2 | 4313 | up regulation | ELISA | 129 Patients/50 NC | 21212676 |  |
| MMP9 | 4318 | up regulation | ELISA | 129 Patients/50 NC | 21212676 |  |
| MMP3 | 4314 | up regulation | ELISA | 129 Patients/90 NC | 21212676 |  |
| KCNA4 | 3739 | up regulation | ELISA | 116 Patients/90 NC | 18262287 |  |
| TNFSF13B | 10673 | up regulation | ELISA | 43 Patients/48 NC | 18852352 |  |
| AGER | 177 | down regulation | ELISA | 42 Patients/36 NC | 22405771 |  |
| NGF | 4803 | up regulation | ELISA | 13 Patients/5 NC | 15763921 |  |
| IGF1 | 3479 | up regulation | quantitative evaluation | 14 Patients/6 NC | 18254780 |  |
| IGF1R | 3480 | up regulation | quantitative evaluation | 14 Patients/6 NC | 18254780 |  |
| TFRC | 7037 | up regulation | Immunogluorescent | 47 Patients/35 NC | 10593570 |  |
| BCL2 | 596 | up regulation | Immunocytochemistry | 10 Patients/4 NC | 8619530 |  |
| BAX | 581 | up regulation | Immunohistochemistry | 38 Patients/5 NC | 11574213 |  |
| MKI67 | 4288 | up regulation | Immunohistochemistry | 38 Patients/5 NC | 11574213 |  |
| IL18 | 3606 | up regulation | ELISA | 78 Patients/33 NC | 12136075 |  |
| CTLA4 | 1493 | up regulation | PCR-RFLP | 208 Patients/173 HC | 16178018 |  |
| CHRNA1 | 1134 | up regulation | RT-PCR | 180 Patients/151 NC | 17687331 |  |
| CHRNB1 | 1140 | up regulation | RT-PCR | 27 Patients/10 NC | 9649579 |  |
| CHRND | 1144 | up regulation | Genotyping | 444 Patients/168 NC | 14735155 |  |
| CHRNE | 1145 | up regulation | qRT-PCR | 27 Patients /10 NC | 9649579 |  |
| CTSL2 | 1515 | down regulation | RT-PCR | 83 Patients/244 NC | 17869649 |  |
| FCGR2A | 2212 | up regulation | allele-specific-PCR | 107 Patients/239 NC | 14597109 |  |
| APOE | 348 | up regulation | PCR-RFLP | 120 Patients/120 NC | 20644276 |  |
| ADRB2 | 154 | up regulation | allele-specific PCR | 145 Patients/96 NC | 10606977 |  |
| IFNG | 3458 | down regulation | PCR–SSP | 115 Patients/204 NC | 17509455 |  |
| IL10 | 3586 | up regulation | PCR | 64 Patients/87 NC | 19299022 |  |
| IL1A | 3552 | positive associated3552 | PCR-RFLP | 421 Patients/995 NC | 11777547 |  |
| IL2RB | 3560 | positive association | TaqMan Allelic Discrimination Assays | 146 Patients/291 NC | 20728947 |  |
| IL4R | 3566 | up regulation | TaqMan Allelic Discrimination Assays | 260 Patients/299 NC | 22119518 |  |
| LGALS1 | 3956 | positive association | TaqMan Allelic Discrimination Assays | 146 Patients/291 NC | 20728947 |  |
| HSP90B1 | 7184 | down regulation | ELISA | 341 Patients/28 NC | 21774995 |  |
| CA3 | 761 | down regulation | Immunoblot assay | 28 Patients/24 NC | 19301202 |  |
| IL12B | 3593 | down regulation | ELISA | 75 Patients/50 NC | 18054287 |  |
| IL12A | 3592 | down regulation | ELISA | 75 Patients/50 NC | 18054287 |  |
| TNIP1 | 10318 | positive association | GWAS | 649 Patients/ 2600 NC | 23055271 |  |
| STAT4 | 6775 | positive association | GWAS | 649 Patients/ 2600 NC | 23055271 |  |
| IKZF1 | 10320 | positive association | GWAS | 649 Patients/ 2600 NC | 23055271 |  |
| IRF5 | 3663 | positive association | GWAS | 649 Patients/ 2600 NC | 23055271 |  |
| NKX2-3 | 159296 | positive association | GWAS | 649 Patients/ 2600 NC | 23055271 |  |
| ORMDL3 | 94103 | positive association | GWAS | 649 Patients/ 2600 NC | 23055271 |  |
| CD226 | 10666 | positive association | GWAS | 649 Patients/ 2600 NC | 23055271 |  |
| PTTG1 | 9232 | positive association | GWAS | 649 Patients/ 2600 NC | 23055271 |  |
| FOXP3 | 50943 | negative association | PCR-RFLP | 118 Patients/124 NC | 23228687 |  |
| DNMT3B | 1789 | positive association | PCR-RFLP | 324 Patients/735 NC | 24260492 |  |
| TLR3 | 7098 | positive association | Flow Cytometry | 35 Patients/9 NC | 23280437 |  |
| EIF2AK2 | 5610 | positive association | Flow Cytometry | 35 Patients/9 NC | 23280437 |  |
| HNMT | 3176 | positive association | TaqMan SNP Genotyping Assay | 213 Patients/342 NC | 23932992 |  |
| CR2 | 1380 | positive association | Flow cytometry | 20 Patients/20 NC | 23266128 |  |
| IL2RA | 3559 | positive association | RT-PCR | 34 Patients/6 NC | 23397576 |  |
| CCL17 | 6361 | positive association | Immunohistochemical analysis | 28 Patients/9 NC | 24397961 |  |
| CCL22 | 6367 | positive association | Immunohistochemical analysis | 28 Patients/9 NC | 24397961 | |
| CCR4 | 1233 | positive association | Immunohistochemical analysis | 28 Patients/9 NC | 24397961 |  |
| IFNL2 | 282616 | positive association | RT-PCR | 31 Patients/30 NC | 24393484 |  |
| HSPA5 | 3309 | positive association | RT-PCR | 13 Patients/5 NC | 24882382 |  |
| IL21 | 59067 | up regulation | RT-PCR | 32 Patients/19 NC | 24518487 |  |
| IL21R | 50615 | positive association | ELISA | 20 Patients/15 NC | 23708911 |  |
| AIRE | 326 | negative association | RT-PCR | 39 Patients/21 NC | 24768600 |  |
| IL15 | 3600 | positive association | multiplexed fluorescent magnetic bead-based immunoassay | 47 Patients/20 NC | 24666229 |  |
| VEGFA | 7422 | positive association | multiplexed fluorescent magnetic bead-based immunoassay | 47 Patients/20 NC | 24666229 |  |
| CXCL8 | 3576 | positive association | multiplexed fluorescent magnetic bead-based immunoassay | 47 Patients/20 NC | 24666229 |  |
| CCL11 | 6356 | positive association | multiplexed fluorescent magnetic bead-based immunoassay | 47 Patients/20 NC | 24666229 |  |
| CCL3 | 6348 | positive association | multiplexed fluorescent magnetic bead-based immunoassay | 47 Patients/20 NC | 24666229 |  |
| CCL4 | 6351 | positive association | multiplexed fluorescent magnetic bead-based immunoassay | 47 Patients/20 NC | 24666229 |  |
| FBCL6 | 604 | up regulation | Western blot | 32 Patients/19 NC | 24518487 |  |
| ICOS | 29851 | up regulation | Western blot | 32 Patients/19 NC | 24518487 |  |
| HAVCR1 | 26762 | up regulation | PCR-SSP | 58 Patients/62 NC | 24959269 |  |
| HLA-DRA | 3122 | up regulation | Fluidigm SNPtype Assays | 1177 Patients/ 814 NC | 25356403 |  |
| AKAP12 | 9590 | up regulation | Fluidigm SNPtype Assays | 1177 Patients/ 814 NC | 25356403 |  |
| CD86 | 942 | up regulation | Fluidigm SNPtype Assays | 1177 Patients/ 814 NC | 25356403 |  |
| VAV1 | 7409 | up regulation | Fluidigm SNPtype Assays | 1177 Patients/ 814 NC | 25356403 |  |
| HMGB1 | 3146 | up regulation | ELISA | 60 Patients/40 NC | 25344065 |  |
| TNFRSF11A3 | 8792 | up regulation | Taq-man genotype assays | 1032 Patients/ 1998 NC | 25643325 |  |
| CSF2 | 1437 | up regulation | ELISA | 11patients/11NC | 26826242 |  |
| TLR7 | 51284 | up regulation | RT-PCR | 26patients/10NC | 26723518 |  |
| TLR9 | 54106 | up regulation | RT-PCR | 26patients/10NC | 26723518 |  |
| IL25 | 64806 | up regulation | ELISA | 32patients/28NC | 26845056 |  |
| IL27 | 246778 | up regulation | quantitative sandwich enzyme immunoassay | 32patients/50NC | 26531704 |  |
| LRP4 | 4038 | positive association | Flow cytofluorimetry | 101patients/85NC | 26284792 |  |
| PRDX5 | 25824 | up regulation | ELISA | 40patients/40NC | 26439955 |  |
| TSLP  IL7 | 85480 | down regulation | qRT-PCR | 52patients/23NC | 26531692 |  |
|  | 3574 | up regulation | ELISA | 22patients/20NC | 25962782 |  |
| TNFAIP8L2 | 79626 | down regulation | qRT-PCR | 25patients/22NC | 26500105 |  |
