# Supplementary material for: Construction of a TF–miRNA–gene feed-forward loop network predicts biomarkers and potential drugs for myasthenia gravis
Source: Sci Rep. 2021 Jan 28;11:2416. doi: 10.1038/s41598-021-81962-6 (PMC7843995; doi:10.1038/s41598-021-81962-6)
Supplement: Supplementary file 8 — Supplementary Table 5. [file 41598_2021_81962_MOESM8_ESM.docx]

**Table S5 Detail information of 128 MG risk miRNAs.**

| **miRNA name** | **Expression** | **PubMed ID** |
| --- | --- | --- |
| hsa-let-7e-5p | down-regulated | 24637658 |
| hsa-miR-30e-5p | down-regulated | 25356381 |
| hsa-let-7c-5p | down-regulated | 23196978 |
| hsa-let-7b-3p | up-regulated | 24734107 |
| hsa-let-7f-1-3p | up-regulated | 24734107 |
| hsa-let-7b-5p | down-regulated | 23196978 |
| hsa-let-7a-5p | down-regulated | 23196978 |
| hsa-let-7d-5p | down-regulated | 23196978 |
| hsa-let-7i-5p | down-regulated | 23196978 |
| hsa-let-7f-5p | down-regulated | 23196978 |
| hsa-let-7g-5p | down-regulated | 22835429 |
| hsa-miR-10a-5p | down-regulated | 24734107 |
| hsa-miR-15b-5p | down-regulated | 23196978 |
| hsa-miR-15a-5p | down-regulated | 22835429 |
| hsa-miR-18b-3p | up-regulated | 24734107 |
| hsa-miR-16-5p | down-regulated | 23196978 |
| hsa-miR-17-5p | down-regulated | 24734107 |
| hsa-miR-20b-5p | down-regulated | 23196978 |
| hsa-miR-20a-5p | down-regulated | 23196978 |
| hsa-miR-21-5p | up-regulated | 25356381 |
| hsa-miR-23a-3p | up-regulated | 22835429 |
| hsa-miR-21-5p | up-regulated | 22835429 |
| hsa-miR-22-5p | down-regulated | 24734107 |
| hsa-miR-24-3p | down-regulated | 24637658 |
| hsa-miR-25-3p | down-regulated | 23196978 |
| hsa-miR-27a-3p | up-regulated | 22835429 |
| hsa-miR-26a-5p | up-regulated | 22835429 |
| hsa-miR-29a-3p | up-regulated | 23196978 |
| hsa-miR-29b-3p | up-regulated | 23196978 |
| hsa-miR-29c-3p | up-regulated | 24637658 |
| hsa-miR-30a-5p | down-regulated | 24734107 |
| hsa-miR-33b-3p | up-regulated | 24734107 |
| hsa-miR-34a-5p | up-regulated | 25356381 |
| hsa-miR-92a-3p | down-regulated | 23196978 |
| hsa-miR-92b-3p | up-regulated | 24734107 |
| hsa-miR-93-5p | down-regulated | 23196978 |
| hsa-miR-107 | down-regulated | 22835429 |
| hsa-miR-106b-3p | up-regulated | 25356381 |
| hsa-miR-106b-5p | down-regulated | 24734107 |
| hsa-miR-122-5p | down-regulated | 24637658 |
| hsa-miR-125a-3p | down-regulated | 24734107 |
| hsa-miR-125b-5p | down-regulated | 24734107 |
| hsa-miR-125a-5p | up-regulated | 24637658 |
| hsa-miR-130b-3p | up-regulated | 24637658 |
| hsa-miR-129-1-3p | up-regulated | 23196978 |
| hsa-miR-129-2-3p | up-regulated | 23196978 |
| hsa-miR-140-3p | down-regulated | 24637658 |
| hsa-miR-139-5p | down-regulated | 22835429 |
| hsa-miR-142-3p | up-regulated | 22835429 |
| hsa-miR-140-5p | down-regulated | 24637658 |
| hsa-miR-142-5p | up-regulated | 24734107 |
| hsa-miR-144-3p | down-regulated | 22835429 |
| hsa-miR-144-5p | down-regulated | 24734107 |
| hsa-miR-145-5p | down-regulated | 24043548 |
| hsa-miR-146a-5p | up-regulated | 24036458 |
| hsa-miR-149-5p | up-regulated | 24734107 |
| hsa-miR-150-5p | up-regulated | 23196978 |
| hsa-miR-155-5p | up-regulated | 24387321 |
| hsa-miR-181a-2-3p | down-regulated | 24734107 |
| hsa-miR-181b-5p | down-regulated | 24734107 |
| hsa-miR-181c-5p | down-regulated | 25962782 |
| hsa-miR-183-5p | down-regulated | 24734107 |
| hsa-miR-185-5p | down-regulated | 23196978 |
| hsa-miR-188-5p | down-regulated | 24734107 |
| hsa-miR-191-3p | up-regulated | 24734107 |
| hsa-miR-193a-3p | down-regulated | 24734107 |
| hsa-miR-192-5p | down-regulated | 24637658 |
| hsa-miR-193a-5p | up-regulated | 24734107 |
| hsa-miR-197-3p | up-regulated | 23196978 |
| hsa-miR-199a-3p | down-regulated | 24637658 |
| hsa-miR-200c-3p | down-regulated | 24734107 |
| hsa-miR-210-3p | down-regulated | 24734107 |
| hsa-miR-221-3p | down-regulated | 24637658 |
| hsa-miR-296-5p | up-regulated | 25356381 |
| hsa-miR-320a | down-regulated | 23196978 |
| hsa-miR-320b | down-regulated | 23196978 |
| hsa-miR-320d | down-regulated | 24734107 |
| hsa-miR-324-3p | down-regulated | 22835429 |
| hsa-miR-331-3p | down-regulated | 24637658 |
| hsa-miR-338-3p | up-regulated | 24734107 |
| hsa-miR-342-3p | up-regulated | 24734107 |
| hsa-miR-345-5p | down-regulated | 24637658 |
| hsa-miR-363-3p | up-regulated | 25356381 |
| hsa-miR-362-5p | down-regulated | 24734107 |
| hsa-miR-365a-3p | up-regulated | 23196978 |
| hsa-miR-375 | down-regulated | 24637658 |
| hsa-miR-421 | up-regulated | 25356381 |
| hsa-miR-423-3p | down-regulated | 24734107 |
| hsa-miR-424-5p | up-regulated | 25356381 |
| hsa-miR-425-5p | up-regulated | 24637658 |
| hsa-miR-451a | down-regulated | 22835429 |
| hsa-miR-486-5p | down-regulated | 23196978 |
| hsa-miR-494-3p | up-regulated | 23196978 |
| hsa-miR-500a-3p | down-regulated | 24734107 |
| hsa-miR-505-5p | down-regulated | 24734107 |
| hsa-miR-518d-3p | up-regulated | 24637658 |
| hsa-miR-520d-5p | up-regulated | 24637658 |
| hsa-miR-523-3p | down-regulated | 24637658 |
| hsa-miR-532-5p | down-regulated | 24734107 |
| hsa-miR-548c-3p | up-regulated | 24637658 |
| hsa-miR-548a-3p | up-regulated | 24637658 |
| hsa-miR-563 | up-regulated | 24734107 |
| hsa-miR-564 | down-regulated | 24734107 |
| hsa-miR-574-3p | down-regulated | 24734107 |
| hsa-miR-584-5p | down-regulated | 24734107 |
| hsa-miR-602 | up-regulated | 23196978 |
| hsa-miR-634 | up-regulated | 23196978 |
| hsa-miR-629-5p | down-regulated | 24734107 |
| hsa-miR-652-3p | down-regulated | 24637658 |
| hsa-miR-665 | up-regulated | 24734107 |
| hsa-miR-664a-5p | down-regulated | 24734107 |
| hsa-miR-766-3p | up-regulated | 24734107 |
| hsa-miR-885-5p | down-regulated | 24637658 |
| hsa-miR-933 | up-regulated | 23196978 |
| hsa-miR-940 | up-regulated | 24734107 |
| hsa-miR-1225-5p/3p | up-regulated | 23196978 |
| hsa-miR-1228-3p | up-regulated | 24734107 |
| hsa-miR-1234-3p | up-regulated | 24734107 |
| hsa-miR-1237-3p | up-regulated | 24734107 |
| hsa-miR-1238-3p | up-regulated | 24734107 |
| hsa-miR-1249-3p | up-regulated | 24734107 |
| hsa-miR-1260a | up-regulated | 23196978 |
| hsa-miR-1267 | up-regulated | 24734107 |
| hsa-miR-1281 | up-regulated | 24734107 |
| hsa-miR-1470 | up-regulated | 24734107 |
| hsa-miR-1539 | up-regulated | 24734107 |
| hsa-miR-1825 | up-regulated | 24734107 |
| hsa-miR-548k | down-regulated | 29661539 |
