# Supplementary material for: Construction of a TF–miRNA–gene feed-forward loop network predicts biomarkers and potential drugs for myasthenia gravis
Source: Sci Rep. 2021 Jan 28;11:2416. doi: 10.1038/s41598-021-81962-6 (PMC7843995; doi:10.1038/s41598-021-81962-6)
Supplement: Supplementary file 9 — Supplementary Table 6. [file 41598_2021_81962_MOESM9_ESM.docx]

**Table S6 Information about MG related transcription factors (TFs).**

| **TF** | **Source Databases** | | |
| --- | --- | --- | --- |
| AIRE |  |  | TRRUST |
| BCL6 | TRED | CHIPbase | TRRUST |
| ECD |  |  | TRRUST |
| EIF2AK2 |  |  | TRRUST |
| ESR1 | TRED | CHIPbase | TRRUST |
| ESR2 | TRED | CHIPbase | TRRUST |
| FOSL1 |  | CHIPbase | TRRUST |
| FOXP3 |  | CHIPbase | TRRUST |
| IKZF1 |  | CHIPbase | TRRUST |
| IRF4 |  | CHIPbase | TRRUST |
| IRF5 |  | CHIPbase | TRRUST |
| IRF8 |  | CHIPbase | TRRUST |
| MAX |  | CHIPbase | TRRUST |
| MYC | TRED | CHIPbase | TRRUST |
| MYOG |  |  | TRRUST |
| NKX2-3 |  | CHIPbase | TRRUST |
| NR3C1 |  | CHIPbase | TRRUST |
| NR4A3 |  |  | TRRUST |
| PTTG1 |  |  | TRRUST |
| STAT4 | TRED | CHIPbase | TRRUST |
| TNFAIP3 |  |  | TRRUST |
